# On the Famous Traditional Chinese Medicine “Fu Zi”: Discovery, Research, and Development of Cardioactive Constituent Mesaconine

**DOI:** 10.1007/s13659-020-00266-w

**Published:** 2020-09-22

**Authors:** Feng-Peng Wang, Ruo-Bing Chao

**Affiliations:** Department of Chemistry of Medicinal Natural Products, West China College of Pharmacy, Sichuan University, Chengdu, 610041 People’s Republic of China

**Keywords:** Fu Zi, *Aconitum carmichaelii*, Diterpenoid alkaloid, Mesaconine

## Abstract

**Abstract:**

This review summarizes the process of the discovery, research, and development of a cardioactive component, mesaconine, from the lateral roots of *Aconitum carmichaelii* (“Fu Zi”). To date, pre-clinical showed that mesaconine is a novel type of cardiotonic lead drug with relatively high potency, low toxicity, and a new mechanism.

**Graphic Abstract:**

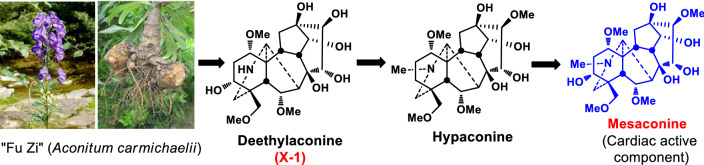

## Introduction

**Fig. 1:**
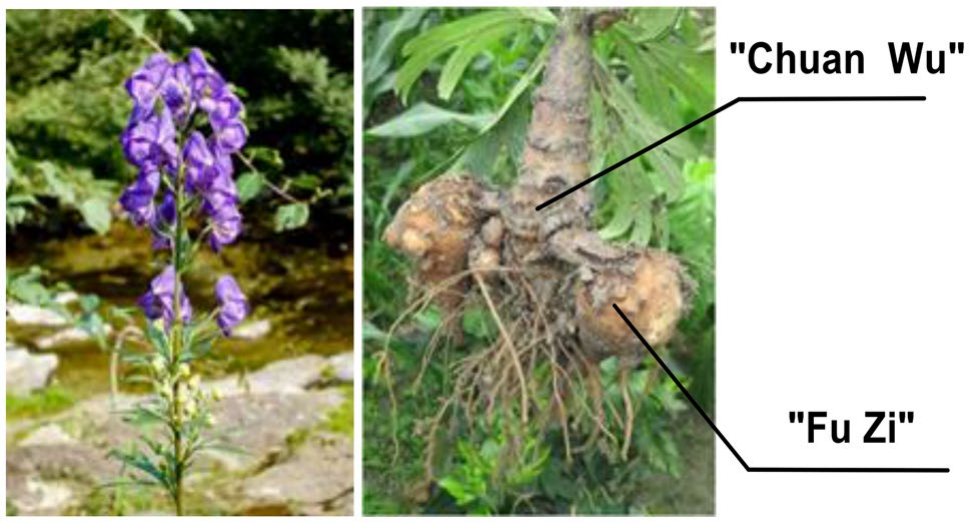
The lateral roots of *A. carmichaelii*

**Fig. 2:**
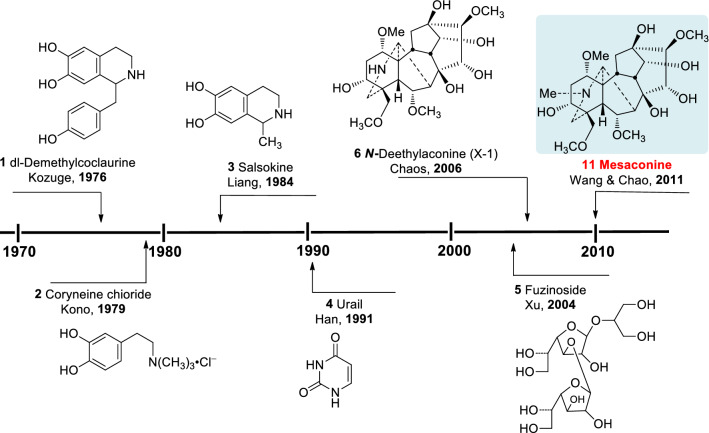
Known cardioactive ingredients of “Fu Zi”

“Fu Zi” is a Chinese medicine derived from the lateral roots of *Aconitum carmichaelii* Dexb. (Ranunculaceae) (Fig. 1) [1]. Endemic to the Jiangyou district in Sichuan Province of China, the plant has now been widely cultivated in Hanzhong of Shanxi Province, and Li Jiang of Yunnan Province, besides the Jiangyou and Butuo areas in Sichuan. “Fu Zi” is a well-known traditional Chinese medicine with a history of clinical applications for more than 2000 years [2]. The use of “Fu Zi” as a cardiotonic drug in China, Japan, and Korea is supported by vast amounts of clinical records and literatures [2, 3]. The search for cardioactive constituents has attracted the attention of many medicinal chemists for several decades. Historically, cardioactive compounds such as dl-demthylcoclaurine (**1**) [4], coryneine chloride (**2**) [5], salsaline (**3**) [6], uracil (**4**) [7] and fuzinoside (**5**) [8] were discovered before 2004 (Fig. 2). However, these compounds are not the key constituents of “Fu Zi”.

dl-Demethylcoclaurine (**1**) was first isolated from the roots of *A. japonicum*, and it is subsequently developing by the Institute of Materia Medica, Chinese Academy of Medical Sciences as a diagnostic agent for coronary heart disease with little cardiac effect [9]. On the other hand, coryneine chloride (**2**) and salsaline (**3**) only exhibit weak cardiac activities, whereas the contents of uracil (**4**) and fuzinoside (**5**) are extremely low in plants. Notably, uracil (**4**) only displayed weak cardiac activity in anesthetized rats [9]. In 2014 we reported that the synthetic fuzinoside had no cardiac activity in the isolated bullfrog heart experiments [10], and its ^13^C NMR data did not match those of fuzinoside reported in the literature [8], showing that the originally proposed structure of fuzinoside (**5**) was incorrect [10].

## Results and Discussion

### Discovery

In the spring of 1979 when I (F.P. Wang) was a graduate student, one of my classmates who worked in the Liangshan district of Sichuan province, told me that one *Aconitum* plant was effective in treating aconitine poisoning. This piece of information attracted the attention of my advisors, Professors Qi-cheng Fang and De-quan Yu. Later, denudatine was isolated from 40 g of the *Aconitum* sample that was sent from my classmate. Animal experiments showed that both denudatine and the total alkaloid fraction had a significant effect of preventing aconitine poisoning. Since then, I started the long journey of studying diterpenoid alkaloids throughout my entire career.

After my post-doctoral research with Professor S. William Pelletier at the Georgia University in 1987, I started the studies of diterpenoid alkaloids independently. Since, at that time, a senior professor in our department was already working on “Fu Zi”, I did not get involved in “Fu Zi” research until 2007.

The story of my research on “Fu Zi” began when a graduate student (Xiu-xiu Liu) from Professor Ruo-bing Chao’s group found a compound (X-1) that displayed cardiac activity. After my careful analysis of its NMR spectra, the structure was elucidated to be *N*-deethylaconine (**6**) [11]. Then Professor Chao sent me a copy of Liu’s doctoral thesis. Attracted by the interesting cardiac activity of compound X-1, I suggested to Professor Chao to remove the structure of X-1 from Liu’s thesis under the consideration of future patent applications. Realizing the low content of X-1 in “Fu Zi” and the tedious isolation process, I started to plan for semi-synthesis immediately, leading to a trial of synthetic work in May 2008. The studies turned out to be much more difficult than I could imagine. In the beginning, we tried to synthesize X-1 through a series of reactions starting from the abundant diterpenoid alkaloid yunaconitine (**7**), but such an attempt failed. Then we turned to aconitine (**8**) as starting material, with an attempt to obtain *N*-deethylaconine through sequential *N*-deethylation and hydrolysis. To our disappointment, the ^13^C NMR spectra of the synthetic product were inconsistent with those of the natural X-1. More importantly, the synthetic product had no cardiac activity. We then focused our attention on the use of mesaconitine (**9**) as starting material, with the hope of producing *N*-deethylaconine by removal of the *N*-methyl (by nitrogen oxidation–reduction) and hydrolysis. Again disappointedly, the NMR spectra of the synthetic product did not match those of the natural X-1. What was the problem? We suspected that there might be a configuration change of some carbons during the synthesis process, albeit unlikely based on our organic chemistry knowledge. In order to verify this hypothesis, the synthesized compound was acetylated to afford single crystals for X-ray crystallographic analysis. The results demonstrated that the configuration of the compound had not been changed.

We then redesigned the reaction sequence, starting from hydrolysis first, and followed by *N*-demethylation. However, the NMR spectra of the product still did not match those of the natural X-1. Finally, mesaconitine (**9**) was taken as the starting material, followed by hydrolysis using sodium hydroxide/methanol and removal of *N*-methyl through nitrogen oxidation–reduction. By employing these milder reactions compared to the previous conditions, we finally succeeded in obtaining a product that displayed the same NMR data as those of the natural X-1 (deethylaconine) (**6**) on June 29th 2009. However, to our surprise, the cardiac activity of the product was only 28%, far less than the observed 110% of the reference compound [12].

**Fig. 3:**
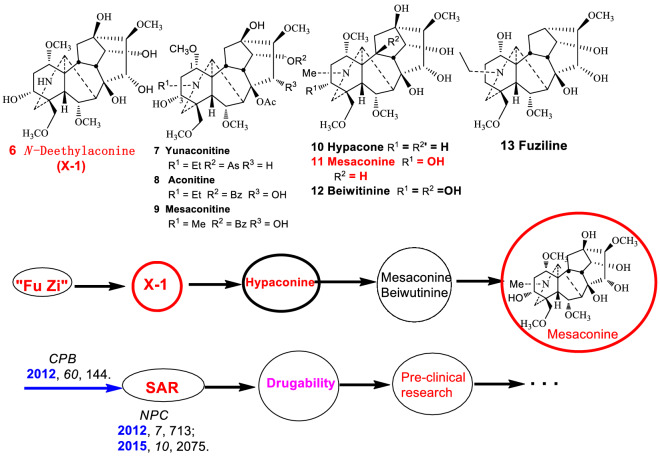
Discovery, research, and development of cardiotonic component mesaconine (**11**) from “Fu Zi”

At this point, two questions had to be answered. First, why compound X-1 was difficult to be synthesized. Second, why the activity of the synthesized compound was much weaker than that of the reference compound. To answer the first question, we proposed that X-1 was unstable, and multiple conformers could be formed during the synthesis. Indeed, careful analysis of the ^13^C NMR spectra revealed the presence of at least 10 conformers [12]. Such a phenomenon is rare in the diterpenoid alkaloids, and even in the alkaloid chemistry. For the second question, we suspected that the reference compound was impure, although TLC analysis displayed only a single spot. We thus decided to measure the NMR spectra of the reference sample. At that moment, we realized that there was little sample of X-1 left behind. After trying very carefully to wash the sample vial with methanol, only 0.6 mg of the reference compound was recovered. Subsequently, the X-1 reference compound was proved to be a mixture, the major component of which was hypaconine (**10**) based on 2D NMR analysis (at 600 MHz). We further confirmed the cardiac activity of hypaconine (**10**). Because of the significant activity observed in hypaconine (**10**), we hypothesized that mesaconine (**11**) and beiwutinine (**12**) present in the same plant family would likely be active as well. The results of isolated bullfrog heart cardioactivity test fully confirmed our hypothesis. Among the four compounds, X-1 was unstable and difficult to prepare; the source of beiwutinine (**12**) was rare and could not be prepared on a large scale, whereas both hypaconine (**10**) and mesaconine (**11**) could be easily prepared. Therefore, the latter two compounds were sent to Professor Xiao-liang Wang, director of the Institute of Materia Medica, Chinese Academy of Medical Sciences, for further pharmacological evaluation in rat models. About half year later, encouraging results arrived. Both compounds showed significant cardiotonic activities, hypaconine (**10**) being not as long-acting as mesaconine (**11**) [13, 14]. This observation was later confirmed by the structure–activity relationship results [15], and we concluded that mesaconine (**11**) was the main cardiotonic component in “Fu Zi”, which have been pursued by medicinal chemists for several decades (Fig. 3).

### Research

After confirming the cardiotonic effect of mesaconine (**11**), we started to plan for structure-activity relationship studies of C_19_-diterpenoid alkaloids. It was realized that the natural contents of both mesaconine (**11**) and its parent alkaloid mesaconitine (**9**) are low in “Chuan Wu” and “Fu Zi”, and the content of aconitine (**8**) is even lower. It became obvious that neither “Chuan Wu” nor “Fu Zi” was feasible sources to obtain mesaconitine (**9**) and its hydrolyzed product mesaconine (**11**). Fortunately, we incidentally found a related species of “Cao Wu”, *A*. *soongaricum*, grown in Xinjiang province, in the traditional Chinese medicine market at Chengdu in July 2009. The plant material contained a high content of aconitine (**8**), up to 1%*.* It was not only rich in aconitine (**8**), but also easy to cultivate. It can be said without exaggeration that *Aconitum soongoricum* was the big savior of this project!

We then moved on to perform structure–activity relationship studies. To summarize the key findings, structure–activity relationship (SAR) data indicated that the cardiotonic activity is strengthened by: (1) an aconitine-type aminoalcohol structure; (2) the presence of 8, 15α-, and 3α-OH groups; and (3) the presence of N-Me or NH groups. Those diterpenoid alkaloids containing *N*-ethyl group such as 15α–hydroxyneoline (fuziline) (**13**) were ineffective, weak or short-lived [15]. An in-depth study in 2015 further revealed that, in addition to the ester group-containing aconitine-type alkaloids, the lycoctonine-type and C_20_-diterpenoid alkaloids had no cardiac activities. It was also noted that the hydrobromide salt of mesaconine (**11**) was ineffective in the isolated bullfrog hearts test [15], but it exhibited positive in vivo activity in rats, despite a slower onset of drug action than the free base mesaconine [16].

From 2012 to 2015, we investigated the drugability of mesaconine (**11**) based on the above results. Notably, a study conducted by Professor Zhuo-wei Hu of the Institute of Materia Medica, Chinese Academy of Medical Sciences, using a mouse adriamycin-induced chronic heart failure model revealed encouraging results in 2015 [17]. In addition, the single maximum tolerated dose in rats, obtained by the Chengdu Chinese Medicine Safety Evaluation Center, reached 1200 mg/kg [12]. All these results paved the way for further research and development of mesaconine (**11**).

### Development

At a regional academic conference in September 2014, our presentation of the research on cardiac activity of “Fu Zi” attracted the attention of Chairman Fu-neng Geng of the Gooddoctor Pharmaceutical Group, a well-known pharmaceutical company in Sichuan province. Chairman Geng has long devoted to the research and production of the traditional medicine *Periplaneta americana* and “Fu Zi”. In the spring of 2016, a research data transfer contract was signed with the Gooddoctor Pharmaceutical Group for the development of the anti-heart failure candidate drug mesaconine (**11**). While the drug development process involves many aspects of pharmaceutical and biological sciences, only part of the production process is discussed herein.

**Fig. 4:**
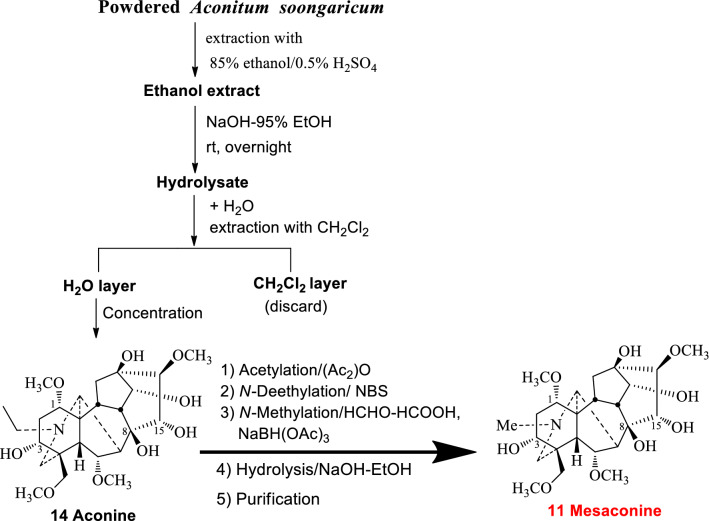
Industrial preparation process for Mesaconine (**11**)

 The semi-synthetic route for the production of mesaconine (**11**) in 2010–2015 was as follows: aconitine (**8**) was extracted and purified, as starting material, followed by stepwise *N*-deethylation, *N*-methylation, and hydrolysis. Later, one of my friends from Chengdu Pusi Biological Company expressed great concerns with this route from an industrial production point of view. The reason was that aconitine (**8**) together with the intermediate mesaconitine (**9**) are highly toxic. It was a blow in the head! We therefore had to start developing an alternative synthetic route. As we all know, most diterpenoid alkaloids are readily soluble in organic solvents, but insoluble in water. However, preliminary experiments showed that the aconitine hydrolysate, aconine (**14**), was reasonable soluble in water but insoluble in organic solvents. This anomalous dissolution character helped to simplify our synthetic strategy. Thus, after the total alkaloids were extracted, they were immediately hydrolyzed into non-toxic aconine (**14**) with sodium hydroxide in ethanol at room temperature and concentrated. After adding water and extracted with dichloromethane, aconine (**14**) could be obtained from the aqueous phase in high purity up to > 95%. Subsequent acetylation, *N*-deethylation, *N*-methylation, and hydrolysis provided mesaconine (**11**) (Fig. 4). In summary, the key features of the production process included the following: (1) isolation of aconitine (**8**) from *Aconitum soongaricum*; (2) taking advantage of the water solubility property of aconine (**14**) for purification; (3) efficient *N*-deethylation reaction especially suitable for aconitine-type diterpenoid alkaloids [16], which we accidentally discovered in 1999 [18].

## Conclusions

1. Diligence is the foundation of success. In necessity hides uncertainty. We encountered many opportunities and contingencies during the study of cardiac constituents of “Fu Zi”. Firstly, if Professor Chao had not researched on “Fu Zi”, I might not have the opportunity to study it in my life. Secondly, Had Professor Chao not sending me the thesis of her graduate student, the strong cardiac constituent of “Fu Zi” would have been only known as compound X-1. Thirdly, if compound X-1 isolated by Professor Chao was the unstable and non-cardiac conformer, the cardiac constituent of “Fu Zi” might still remain a mystery. In addition, if my classmate had not raised the topic of aconitine poisoning in 1979, it was likely that I would not get involved in the life-long research of diterpenoid alkaloids. Moreover, the high-efficiency reaction of *N*-deethylation (NBS method) that we accidentally discovered in 1999 [18] had become quite useful in the later stage synthesis of mesaconine (**11**). Even more amazing was that we accidentally found *A. soongaricum* with a high content of aconitine (**8**) in July 2009.
2. In 1934, Wolffe et al. [19] from Temple University in Philadelphia, found that aconine (**14**) had cardiotonic activity in anesthetized dogs. On the contrary, in the 1980s, Chinese scholar Yuan-peng Zhou et al. [20] demonstrated that aconine (**14**) had no cardiac activity in anesthetized cats. On the other hand, when using 1/50 dose of its LD_50_, the compound displayed significant antihypertensive and myocardial toxicity. It is conceivable that, if aconine (**14**) was verified to have cardiac activity at that time, it would have been easily correlated to the relevant alkaloid mesaconine (**11**). In 1956, Professor Goto Tanjiu [21] of the Tokyo Medical College in Japan reported that mesaconine (**11**) had no cardiac activity based on tests using the isolated frog and toad hearts. Nevertheless, Professor Chao was able to show the cardiotonic activity of mesaconine (**11**) in the isolated bullfrog heart model. We are not certain why discrepancies occurred but it seems luck was on our side!
3. The pre-clinical research of mesaconine (**11**) has come to completion. Based on existing results, it can be clearly concluded that mesaconine (**11**) is a novel type of anti-heart failure candidate drug with high potency, low toxicity, and a new mechanism. Investigational New Drug Application (IND) for the compound is expected in the year 2021. We are proud to have brought this research to the current milestone and look forward to seeing further development of this interesting compound into a useful drug in the research of the cardiac active constituents of “Fu Zi” as well!
